# A COST-UTILITY ANALYSIS FOR RETURN-TO-WORK INTERVENTIONS COMPARING ALTERNATIVE METHODS FOR HANDLING MISSING HEALTH-RELATED QUALITY OF LIFE DATA

**DOI:** 10.2340/jrm.v57.42359

**Published:** 2025-12-01

**Authors:** Cindy NGUYEN, Emily A. BURGER, Lene AASDAHL, Niccolò MORGANTE, Marius STEIRO FIMLAND, Gudrun MARIA WAALER BJØRNELV

**Affiliations:** 1 Department of Health Management and Health Economics, University of Oslo, Oslo, Norway; 2 Erasmus University of Rotterdam, Rotterdam, Netherlands; 3 Center for Health Decision Science, Harvard T.H. Chan School of Public Health, Boston, MA, USA; 4 Department of Public Health and Nursing, Norwegian University of Science and Technology, Trondheim, Norway; 5 Unicare Helsefort Rehabilitation Centre, Rissa, Norway; 6 Department of Neuromedicine and Movement Science, Norwegian University of Science and Technology, Trondheim, Norway

**Keywords:** costs and cost analysis, cost-effectiveness analysis, data management, observer variation, data interpretation, statistical, return to work, sick leave

## Abstract

**Objective:**

Perform a cost-utility analysis for return-to-work interventions with missing health-related quality-of-life (HRQoL) data while transparently demonstrating the impact of different methods of handling missing data on outcomes.

**Methods:**

The costs and quality-adjusted life-years over a 2-year period were estimated for 2 return-to-work interventions, inpatient multimodal occupational rehabilitation (I-MORE) and outpatient acceptance and commitment therapy (O-ACT), using a healthcare perspective and a limited societal perspective. Four methods were used to handle the missing HRQoL data: complete case analysis, single imputation, multiple imputation, and linear mixed models. The cost-effectiveness outcomes were expressed as incremental net monetary benefit.

**Results:**

The average incremental quality-adjusted life-years comparing I-MORE with O-ACT ranged between –0.001 and 0.330 depending on missingness method. From a healthcare perspective, I-MORE was consistently not cost-effective (incremental net monetary benefits ranged from –€7,094 to –€9,363) while from a limited societal perspective, I-MORE was consistently cost-effective (incremental net monetary benefits ranged from €1,293 to €16,277).

**Conclusion:**

While cost-effectiveness findings remained consistent within each analytical perspective, the choice of different missingness methods led to variations in incremental quality-adjusted life-years. Multiple imputation is recommended to handle missing HRQoL data as it is transparent and flexible. However, a thorough investigation of the missing data mechanism should still be conducted.

## LAY ABSTRACT

Prior to the implementation of a new health intervention, the costs and health effects of the intervention are compared, typically, with usual care using a cost-effectiveness analysis. The effects are often measured in quality-adjusted life years, where the health-related quality of life is a key input. However, surveys that measure health-related quality-of-life data often suffer from high non-response, especially in interventions tailored to those who are on sick leave. We evaluated the costs and effects of 2 return-to-work interventions, while describing and applying the most common methods used to handle missing health-related quality-of-life data. We found that different methods to handle missingness did not affect the conclusions of the study, but different methods do lead to different incremental effects, thus warranting the use of several methods in applied cost-effectiveness analyses.

Norway and similar countries face a high rate of sick leave, imposing a substantial burden to both the individual and society (1). Accordingly, over the last decades different return to work (RTW) interventions have been developed and evaluated within randomized controlled trials (RCTs) (2–9). As healthcare resources are sparse, cost-effectiveness analyses (CEAs), which simultaneously compare costs and effects of 2 or more interventions, are needed to accompany RCTs to inform decision-makers on whether to implement these RTW interventions (2, 5, 7, 10).

Ideally, cost-utility analyses (CUA) – which are a type of CEA using generic and preference-based measures of health effect, e.g., quality-adjusted life-years (QALYs) – are preferred to enable the comparison of interventions across diverse patient populations and disciplines in health (11). Estimating QALYs relies on questionnaires eliciting health-related quality-of-life (HRQoL). However, these questionnaires frequently have a large amount of missing data because they require direct input from patients at multiple timepoints over the course of a study (12–14). When faced with these missing data, studies may opt to use disease-specific measures of effect rather than QALYs. In a recent study, inpatient multimodal occupational rehabilitation (I-MORE) was found to be cost-effective compared with outpatient acceptance and commitment therapy (O-ACT); however, the health effects were evaluated using *sickness absence days* (15). A similar effect measure was chosen by Finnes et al. in their cost-effectiveness study on RTW interventions (5). While the use of QALYs as an effect measure is preferred, calculating them without sufficient reporting or handling of missingness will potentially bias the CUA results (12, 16).

With no official guidelines or requirements on how missingness should be handled in a CUA, the methods used to deal with missing data vary greatly between CUAs. Some studies opt to remove all individuals with missing values (17), while others use methods that involve imputation, where the missing data of each individual are replaced with a predicted value (4, 18). Regardless of the method used to handle missingness, it is rare that a study provides a description of the amount of missingness or an explanation of the pattern of missingness to justify the decision for choice of imputation model. There have been articles that aim to provide a guideline for handling missingness (12, 19); however, to the best of our knowledge, no studies have included a thorough explanation of how to apply multiple methods to handle missingness for a trial-based CUA for RTW interventions and presented the outcomes of each method.

The objective of this study was to perform a CUA comparing I-MORE with O-ACT using patient-level data from a 24-month follow-up RCT (3). Simultaneously, we aimed to describe and apply multiple methods for handling missing HRQoL data, and report how different methods may impact CUA outcomes. The current analysis extends the previously published CEA done alongside the Hysnes RCT (ClinicalTrials.gov: NCT01926574) that estimated health effects in terms of “days with sickness absence avoided” (15). We provide Stata codes to guide future practice for how to handle missingness in trial-based CUAs.

## METHODS

### Economic evaluation

Economic evaluations assess whether the effects of an intervention justify the costs when compared with another intervention (the comparator) to ensure the efficient use of scarce healthcare resources (11). In a CUA, health effects are captured using QALYs and costs and are measured by identifying, quantifying and valuing resources. We performed a CUA over 24 months under 2 perspectives: (*i*) a healthcare perspective, taking costs of only healthcare resource use into consideration, and (*ii*) a limited societal perspective, where we included costs of lost productivity due to sickness absence. We chose not to discount costs and effects, consistent with the prior CEA (15).

### Population

The population of the Hysnes RCT consisted of 166 individuals between 18 and 60 years of age who had a sick leave status of at least 50% for 2–12 months prior to inclusion due to musculoskeletal, psychological, or general and unspecified disorders as classified by the 2nd edition of the International Classification of Primary Care (ICPC-2) (3). Further details can be found in the trial paper (3).

### Interventions

We compared 2 RTW interventions, I-MORE and O-ACT (3). I-MORE was an inpatient programme that lasted 3.5 weeks and included ACT and RTW-focused elements (7). The comparator, O-ACT, was an outpatient programme which focused on group-based ACT over 6 weekly sessions that lasted 2.5 hours each (7). I-MORE has been shown to result in fewer days of sickness absence at 12 and 24 months of follow-up (3). Further details can be found in the trial paper (3).

### Data

The trial data included information on individuals’ baseline characteristics (age, gender, educational level, and marital status) and outcomes obtained from patient questionnaires, including the 15D questionnaire for HRQoL (7, 20), Hospital Anxiety and Depression Scale (HADS) for anxiety and depression (21), and the Brief Pain Inventory (BPI) for pain (22).

### Healthcare resource use and costs

**Fig. 1:**
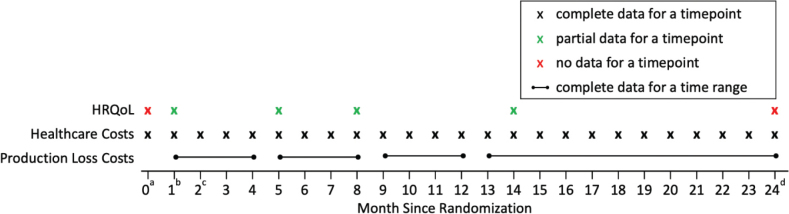
Schematic of the available data at different timepoints. ^a^Baseline. ^b^Programme start assumed to be at month 1. ^c^Both interventions assumed to last 1 month, and programme end is at month 2. ^d^Last follow-up.

The trial data were linked with the Norwegian Health Economics Administration and the Norwegian Patient Registry, providing complete healthcare resource use data for each individual (Fig. 1) (15). Monthly healthcare costs in Norwegian Krone (NOK) included costs for general practitioners (GPs), psychologists, physiotherapists, chiropractors, specialists, somatic healthcare, rehabilitation, and imaging for each individual (Appendix S1; Table SI). Costs were converted to Euro (€) using the average annual 2016 exchange rate (€1 = NOK 9.2899) and converted to May 2024 costs using an inflation rate of 28.9% from Statistics Norway (23, 24). The I-MORE and O-ACT programmes cost €19,628 and €1,531, respectively, applied as a lump sum at the start of the study (15).

### Productivity loss

The data for sickness absence were obtained from the Norwegian National Social Security System Registry and were complete for all individuals in the study (see Fig. 1) (3). We calculated costs of productivity loss by multiplying the number of sickness absence days from work by a fixed cost of absence per day. We assumed a cost of absence per day of €339 (average national wage in 2016 from Statistics Norway [25]) to remain consistent with the published CEA, which we estimated to be €437 after inflating to 2024 (15).

### Health effects

The RCT captured health effects of each intervention using HRQoL, which is measured on a 0–1 scale (0 = death, 1 = perfect health). The 15D questionnaire, which asks questions on 15 dimensions of health, was used to measure HRQoL (7, 20). A valuation algorithm tailored to Norwegians was used to calculate 15D scores for each individual (26).

Patients completed the 15D questionnaire at programme start and at 3, 6, and 12 months after the end of the RTW interventions (see Fig. 1). No questionnaires were administered at baseline (month 0) or at 24-months. For simplicity we assumed the interventions began at 1 month after baseline, both programmes lasted 1 month, and questionnaires were done within the correct follow-up month. Although the programme lengths differed for I-MORE and O-ACT and time of questionnaire completion varied between individuals, the clinical trial results were robust to sensitivity analyses using actual timing. Thus, HRQoL data were collected at 1, 5, 8, and 14 months and completely missing at 0, and 24 months after baseline (Fig. 1).

### Methods to handle missing HRQoL data

There is substantial literature on methods for handling missing data, spanning from simple techniques (e.g., mean imputation) to sophisticated machine learning approaches (27, 28). However, trial-based CUAs pose unique challenges – such as the need to convert longitudinal HRQoL data into QALYs – that require tailored methodological approaches (19, 29). When missingness occurs in multiple inputs, such as both HRQoL and cost data, the complexity of analysis increases. In contrast, our study is distinct in that only HRQoL data are missing, while cost data – including healthcare and productivity loss – are complete. Given this specific missing data pattern and the characteristics of trial-based CUAs, we apply a focused set of established methods drawn from the broader literature that are well suited to our context.

We applied 4 commonly used methods to handle missing HRQoL data: (*i*) complete case analysis (CCA), (*ii*) single imputation (SI), (*iii*) multiple imputation (MI), and (*iv*) linear mixed models (LMM). The first method, CCA, includes only individuals with complete HRQoL data at all timepoints (12, 30). The second method, SI, imputes missing values using simple approaches (mean imputation [12, 31] and last value carried forward [12, 32]) or linear regression, where values are predicted using information from other variables (12, 31). The third method, MI, generates m datasets with imputed values, analyses each separately, then combines estimates into 1 set of outcomes using Rubin’s rules (12, 31). MI can be implemented using chained equations, which predicts missing values based on imputed values of other variables then predictive mean matching (PMM) is used to ensure plausibility by imputing missing values with observed values from individuals with similar predicted values. (12, 31, 33). Finally, LMM predicts values by maximum likelihood estimation using all the available data without any imputation (Appendix S2) (12, 19, 31, 34).

To choose the appropriate method to address missingness, the mechanism by which the data are missing must first be determined (12). If data are missing completely at random (MCAR), complete case analysis (CCA) may be appropriate, as individuals with complete data are assumed representative. If data are missing at random (MAR), the probability of missingness depends only on *observed* and *controllable* variables, such as baseline characteristics. If data are missing not at random (MNAR), we assume that the probability that data are missing depends on unobserved factors, which influences the HRQoL value (12). SI using linear regression, MI, and LMM assume MAR data (12).

### Analyses

We conducted a 2-part analysis. First, we carried out a missing data analysis where we applied each of the 4 methods of handling missingness to estimate mean HRQoL – which was then summarized into QALYs – for I-MORE and O-ACT for all timepoints. Second, we conducted 4 CUAs for each set of estimated QALYs under the 2 analysis perspectives: healthcare perspective and limited societal perspective. All analyses were performed in Stata 18 (StataCorp LLC, College Station, TX, USA) (35). The Stata codes of each method are provided in Appendix S3.

### Statistical analyses for missing data

We first examined missing HRQoL trends to assess the mechanism of missingness, followed by logistic regressions to identify baseline predictors. If the missingness trends varied between interventions and timepoints, and baseline variables were predictors of missingness, we could assume the data were unlikely to be MCAR. We performed linear regressions to determine which variables had an effect (*a* = 0.1) on both the missingness and the values of HRQoL to inform our choice of covariates in our prediction models. The variables that we tested were previous HRQoL, age, gender, education, marital status, disability status, ICPC-2 diagnosis, BPI scores, HADS scores, intervention group, absence from work at each timepoint and cumulative absence up to each timepoint (Appendix S4). To maintain consistency, we used the same chosen covariates for all methods.

We estimated HRQoL at 1, 5, 8, and 14 months after baseline using CCA, SI, MI, and LMM. For the CCA, all individuals missing 15D scores at any follow-up (*n* = 117) were excluded, resulting in a reduced sample size of *n* = 42 (O-ACT = 21, I-MORE = 21). In the second approach using SI, missing HRQoL values were imputed using linear regression predictions based on selected covariates; values exceeding 1 were capped at 1. For MI, we generated 10 imputed datasets (m = 10) using chained equations with predictive mean matching (k = 5), and combined estimates with Rubin’s rules using Stata’s “mi estimate” command to produce a single mean estimate for HRQoL at each of the timepoints (36). Finally, LMM was implemented using a mixed model for repeated measurements, including a time-by-intervention interaction to estimate treatment effects at each timepoint (see Appendix S2) (19). In the LMM method, as there was no imputation involved, HRQoL values were estimated for the whole sample and not at the individual level as in the other methods.

To capture parameter and stochastic uncertainty, we applied each method of handling missingness to 5,000 bootstrapped samples, in which we obtained the mean values of total healthcare costs, total productivity loss costs, estimated HRQoL at each timepoint, and total QALYs for each sample (calculated using the area under the curve method, derived from HRQoL and time) (11). Our 95% credible intervals (CIs) for the mean estimates were produced using the 2.5th and 97.5th percentile of all 5,000 samples.

Before applying the methods to handle missingness, we imputed values at baseline (month 0) for all individuals using the mean HRQoL at 1 month (0.595). Additionally, as all baseline explanatory variables had very few (4 or less) missing values, we imputed the missing values using the median value of the respective variable. After handling missingness at 1, 5, 8, and 14 months, we imputed the completely missing HRQoL data at 24 months. We assumed the treatment effect on HRQoL merged after the HRQoL at 14 months and was maintained until 24 months. As a result, within each missingness method we imputed HRQoL at 15 and 24 months for all individuals using the mean of the estimated HRQoL at 14 months.

### Cost-utility analysis

The final outcome of our CUA was incremental net monetary benefit (INMB) (37). The INMB is calculated as follows:


INMB=ΔQALYs*λ−Δcosts (1)

where ΔQALYs and Δcosts represent the difference in QALYs and costs, respectively, when comparing I-MORE with O-ACT. *λ* represents the cost-effectiveness threshold, which is the maximum cost that is acceptable to pay for a QALY gained. The cost-effectiveness threshold is used as a decision rule when determining whether an intervention is cost-effective (11). The amount that countries are willing to spend for a QALY gain differs. We used a threshold of €30,000 per QALY gained, which is recommended for lower severity diseases in Norway (38). A positive INMB indicates that I-MORE is cost-effective and a negative INMB indicates that it is not cost-effective when compared with O-ACT.

To explore the uncertainty due to the cost-effectiveness threshold, we constructed a cost-effectiveness acceptability curve using results from the bootstrapped analyses (*n* = 5,000). This curve depicts the probability that an intervention is cost-effective across different thresholds (€0 per QALY gained to €100,000 per QALY gained) (11).

### MNAR sensitivity analysis

In an MNAR sensitivity analysis, we adjusted the predicted values to be 10% lower or higher before they were imputed for the SI and MI methods to explore assumptions that those with missing data are different from those without missing values.

### Scenario analyses

For a scenario analysis, we explored different methods for predicting HRQoL at 24 months. We also explored a scenario analysis using a time horizon of 14 months instead of 24 months to align with the CCA time horizon. For further details, see Appendix S5.

## RESULTS

### Baseline characteristics

We included 159 individuals (7 individuals from the trial were excluded due to lack of consent) of whom 81 individuals were in I-MORE and 78 were in O-ACT. Baseline characteristics were similar between the 2 groups (Table I).

**Table I:** Baseline characteristics of participants by intervention

Item	O-ACT (*n* = 78)	I-MORE (*n* = 81)
Age, mean (SD)	45.3 (10.5)	46.4 (8.6)
Gender, *n* (%)		
Male	19 (24%)	16 (20%)
Female	59 (76%)	65 (80%)
Marital status, *n* (%)		
Married	39 (50%)	38 (47%)
Cohabitant	39 (50%)	43 (53%)
Disability, *n* (%)		
No disability	72 (95%)	75 (93%)
Disability	4 (5%)	6 (7%)
Social benefit, n (%)		
100% sick leave	39 (50%)	38 (47%)
Partial sick leave	36 (46%)	41 (51%)
Other	3 (4%)	2 (2%)
International Classification of Primary Care-2 diagnosis, *n* (%)		
A: general and unspecified	9 (12%)	3 (4%)
L: musculoskeletal	39 (50%)	53 (65%)
P: psychiatric	30 (38%)	25 (31%)
Brief Pain Inventory score, mean (SD)		
Average	4.8 (2.1)	4.9 (2.1)
Strongest	6.1 (2.5)	6.5 (1.9)
Hospital Anxiety and Depression Scale, mean (SD)		
Anxiety	8.7 (4.1)	7.3 (3.9)
Depression	6.7 4.0)	5.5 (4.0)

O-ACT: outpatient acceptance and commitment therapy; I-MORE: inpatient multimodal occupational rehabilitation.

### HRQoL

*Descriptive analysis of missingness*. Over the 24-month period, 26% of individuals (*n* = 42) had complete HRQoL data for all 4 timepoints (Appendix S1; Table SII). O-ACT had higher proportions of missingness at most timepoints (except for month 14) compared to I-MORE (Table II). Based on the logistic and linear regressions, the following variables had a significant effect on the probability of missingness and the value of HRQoL: previous HRQoL, age, gender, marital status, ICPC-2 diagnosis, HADS scores, intervention, absence from work at each timepoint and cumulative absence up to each timepoint (see Appendix S4). These variables were therefore chosen as covariates in the prediction models for SI, MI, and LMM. Based on the descriptive analysis of missingness, we assumed that data were MAR, since missingness differed between the 2 interventions over time and was affected by baseline variables.

**Table II:** Number and percentage of individuals with missing HRQoL data at each timepoint by intervention

Months from baseline	O-ACT (*n* = 78)		I-MORE (*n* = 81)		Total (*n* = 159)	
	*n*	% missing	*n*	% missing	*n*	% missing
1 month	48	38%	64	21%	112	30%
5 months	36	54%	45	44%	81	49%
8 months	28	64%	33	59%	61	62%
14 months	31	60%	31	62%	62	61%

O-ACT: outpatient acceptance and commitment therapy, I-MORE: inpatient multimodal occupational rehabilitation.

**Fig. 2:**
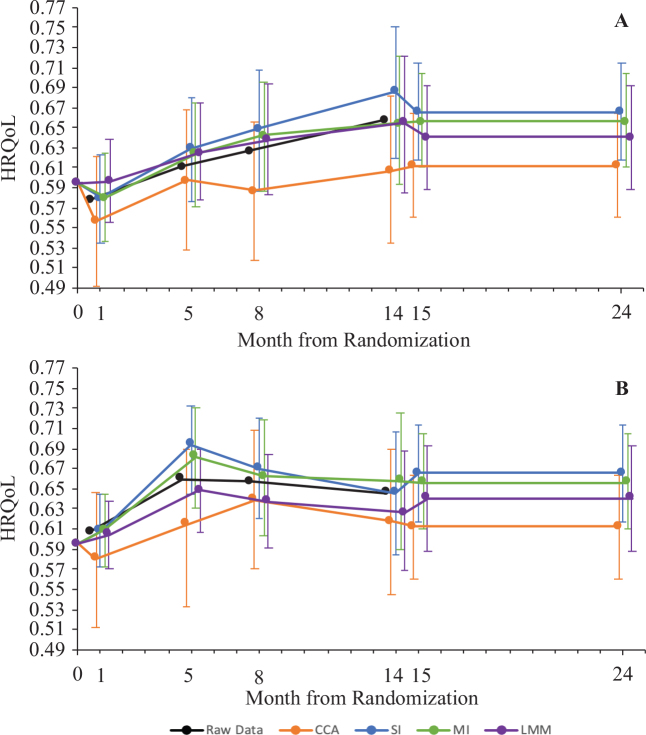
Comparison of mean health-related quality-of-life (HRQoL) over time by intervention for the different methods of handing missing data after 5,000 bootstrap simulations. Data before missingness methods are applied (raw data) are also shown for comparison. Error bars represent 95% confidence bounds, determined by taking the 2.5th percentile and the 97.5th percentile of the bootstrap replications. Note: the scale on the HRQoL axis is condensed to 0.49–0.77 (instead of 0–1) for visibility. (A) Results outpatient acceptance and commitment therapy (O-ACT). (B) Results for inpatient multimodal occupational rehabilitation (I-MORE). CCA: complete case analysis, SI: single imputation, MI: multiple imputation, LMM: linear mixed models.

*Mean HRQoL*. The mean HRQoL values ranged from 0.491 to 0.750 across all timepoints and missingness methods (Appendix S1; Table SIII). For each timepoint, the 95% CIs for the HRQoL estimates overlapped between the different methods with few exceptions (Fig. 2). Notably, the mean HRQoL values over time for the CCA were consistently lower than the other methods, especially in the O-ACT intervention (Fig. 2). Additionally, mean estimated HRQoL for I-MORE was higher than O-ACT at all timepoints, except for SI and LMM, where O-ACT had higher mean HRQoL than I-MORE after month 8 (Appendix S1; Fig S1.1).

### Cost-utility analysis

Over the 24-month study period, total healthcare costs were higher for I-MORE (€25,867, SD: €10,050) compared with O-ACT (€10,344, SD: €14,071). Contrarily, the costs of productivity loss were lower for I-MORE (€112,654, SD: €86,595) compared with O-ACT (€134,431, SD: €85,455); therefore, when productivity loss costs were included under the limited societal perspective, the total cost of I-MORE became €2,649 lower than O-ACT. These results are in line with the previous CEA (15) (see Appendix S1: Table SI).

After 5,000 bootstraps using each missingness method, the incremental QALYs comparing I-MORE with O-ACT ranged between –0.001 (LMM) and 0.33 (CCA) (Table III). Despite this variation, I-MORE was consistently not cost-effective compared with O-ACT (negative expected INMBs) from a healthcare perspective (CCA: –€7,094, SI: –€8,786, MI: –€8,380, LMM: –€9,363). In contrast, I-MORE was consistently cost-effective compared with O-ACT (positive expected INMBs) from a limited societal perspective (CCA: €16,277, SI: €1,875, MI: €2,315, LMM: €1,293) using a cost-effectiveness threshold of €30,000 per QALY gained (Table III). Across all thresholds explored, I-MORE had a lower probability of being cost-effective in the healthcare perspective and a higher probability in the limited societal perspective compared with O-ACT (Appendix S1; Fig S1.2).

**Table III:** Results of using different methods to handle missing health-related quality of life data in a cost-utility analysis of I-MORE vs O-ACT over a 24-month time horizon

Item	CCA^a^	SI	MI	LMM
QALYs O-ACT	1.196	1.300	1.278	1.269
QALYs I-MORE	1.230	1.319	1.310	1.268
Inc QALYs	0.033	0.019	0.032	–0.001
Healthcare perspective				
Costs O-ACT (€)	6,184	6,218	6,240	6,232
Costs I-MORE (€)	14,275	15,577	15,571	15,576
Incremental costs (€)	8,091	9,359	9,331	9,344
INMB^b^ (€)	–7,094	–8,786	–8,380	–9,363
Probability of CE^b^ (%)	0	0	0	0
Limited societal perspective				
Costs O-ACT (€)	77,509	71,785	71,929	71,869
Costs I-MORE (€)	62,229	70,483	70,565	70,558
Incremental costs (€)	–15,280	–1,302	–1,365	–1,311
INMB^b^ (€)	16,277	1,875	2,315	1,293
Probability of CE^b^ (%)	90	61	63	57

Estimates are presented as means after 5000 bootstrap simulations. Costs in 2024 Euros (€).

I-MORE: inpatient multimodal occupational rehabilitation, O-ACT: outpatient acceptance and commitment therapy, CCA: complete case analysis, SI: single imputation, MI: multiple imputation, LMM: linear mixed models, QALY: quality-adjusted life year, INMB: incremental net monetary benefit.

a CCA sample size is 42 instead of 159.

b Based on a cost-effectiveness threshold of €30,000 per QALY gained.

### MNAR sensitivity analysis

After the MNAR sensitivity analysis we found the cost-effectiveness conclusions remained consistent with our base case assumptions within each perspective: INMBs remained negative for the healthcare perspective and positive for the limited societal perspective (Appendix S1; Tables SIV–SV).

### Scenario analyses

The cost-effectiveness outcomes remained robust to all scenario analyses, where INMBs were negative for SI, MI, and LMM in the healthcare perspective and positive in the limited societal perspective, except for the scenario using a 14-month time horizon instead of 24 months (see Appendix S5).

## DISCUSSION

Across the 4 methods that we considered for handling missing HRQoL data, we found important variations in the estimated incremental QALYs. Despite these differences, over a 24-month time horizon I-MORE was consistently not cost-effective compared with O-ACT using a healthcare perspective, but consistently cost-effective once we included costs of productivity loss in a limited societal perspective. To our knowledge, this is the first CUA that applies 4 alternative methods (CCA, SI, MI, and LMM), accompanied by a detailed description of each, to address missing HRQoL data within a RTW trial.

We found notable differences between the methods to handle missingness. First, when predicting mean HRQoL estimates, SI and LMM had a different trend in mean HRQoL overtime from MI and CCA. After month 8, the mean HRQoL for O-ACT, as estimated using the SI and LMM methods, surpassed that of I-MORE. In contrast, under the CCA and MI methods, I-MORE consistently demonstrated higher HRQoL than O-ACT over time. These findings underscore the methodological similarities between SI and LMM in contrast to MI, and illustrate how different approaches to handling missing data can alter observed trends, with potentially significant implications for cost-effectiveness results.

The characteristics of each method also vary, which we took into consideration when determining the recommended method to handle missingness in HRQoL data. The simplest method, the CCA, was the quickest (Appendix S1; Table SVI) to implement and is the most common method used in CEAs even without proof that the data are MCAR (39). We determined our data could not be MCAR in our missing data analysis, rendering the CCA inappropriate. A commonly used alternative is SI using linear regression, which is simple to understand and implement but treats imputed values as if they are the real values, which can underestimate the uncertainty around using imputed values and produce biased estimates (12, 31, 32). In contrast, MI does account for the uncertainty that arises with using imputed values by combining multiple imputed sets, although a disadvantage with MI is that it requires an initial learning curve to understand and implement. MI also required the most computational time, around 41 h compared with SI (6 min) and LMM (1 h 44 min), to execute 5,000 bootstraps in our sample (Appendix S1; Table SVI). The final method, LMM, has the advantage that it reduces the possibility of imputing an unrealistic value because estimates are produced without any imputation (12, 31, 34). One limitation is that LMM does not generate patient-level predictions of HRQoL values, thereby preventing analysis at a disaggregated level and hindering the ability to conduct sensitivity analyses for data that are MNAR (which is possible using SI and MI).

Based on the findings of this study, we believe MI should be considered the gold standard method for handling missing data under the MAR assumption because it offers a transparent approach that appropriately addresses the complexity of imputing repeated HRQoL measures. Moreover, MI is relatively straightforward to implement once the initial methodological learning curve is overcome. However, this recommendation should be interpreted with caution, as the nature of missing data can vary across studies. A thorough analysis and clear description of the missingness mechanism should always precede the selection of a method to handle missingness. Especially if data are deemed MNAR, then no method we used is valid without knowing by how much and in what direction these values should be adjusted.

The main strength of our paper is that it provides an extensive and transparent descriptive analysis of missingness, followed by the application of 4 different methods to handle the missing data to produce HRQoL estimates to be used in a CUA. For each step of the analysis, we provided Stata code along with a detailed explanation of how to apply each missingness method. To our knowledge, there are no other CUAs for RTW interventions that have handled missingness as carefully and transparently as we have. Particularly when an individual was removed from analysis for the CCA, their data that were available – such as their HRQoL at other timepoints and cost data – were also removed. Whereas the SI, MI, and LMM methods had no loss of information because all available data for all 159 participants were included. As a result, the CCA produced HRQoL estimates that were lower (Appendix S1; Table SIII) and incremental costs that were over 10 times greater (see Table III) than when compared with the other methods, indicating that those with complete information differed from the total study population. A plausible explanation for this phenomenon is that those who returned to work, and were therefore better off, did not have time to complete questionnaires at follow-ups. In line with this theory, the logistic regression revealed that those with higher HRQoL at a follow-up were more likely to have a missing 15D questionnaire at the next follow-up (see Appendix S4). Consequently, using the CCA method to handle missingness in a CUA may result in large variations in costs and QALYs such that an intervention may be deemed cost-effective when it is not, and vice versa.

Our study provides a useful example of the types of missing data patterns that are likely to arise in future CUAs. The conventional pattern of missingness typically observed in other CUAs is when data are missing in both outcomes and cost measures. In contrast, our CUA addresses missingness that is isolated to HRQoL data only because we used complete registry data for sickness absence days and healthcare resource use and costs. The World Health Organization highlights the movement towards digitalizing health in many countries and associated benefits, further supporting the expectation that the pattern of missingness observed in our study will become increasingly common in future analyses (40). This increases the relevance of our findings and supports the use of simpler methods for handling missing data in CUAs.

Our study has a few limitations. First, we found that the analytic perspective – specifically, the inclusion of productivity costs – was the primary driver of outcomes, rather than the method used to handle QALY missingness. This underscores the influence of considering productivity loss when informing decision-makers and employers about the cost-effectiveness of RTW interventions. This finding is particularly relevant for countries like Norway, where there is ongoing debate over which perspective should be adopted (41–43). Those in favour of keeping the extended healthcare perspective argue that societal costs can lead to inequality because it can prioritize interventions that target diseases which affect only those who are employed, such as RTW interventions (44). In the meantime, reporting both the healthcare and societal perspective allows for the most information.

Regarding missingness, the percentage of missing HRQoL data in our CUA was quite substantial at 74%. This level of missingness is far above what is recommended for imputation, as such a high degree of missingness inevitably increases bias (12, 45). Consequently, performing the CUA may be inappropriate due to the significant uncertainty in the missing values; therefore our reported HRQoL and QALYs of I-MORE and O-ACT should be interpreted with caution. Nevertheless, we believe that the analyses are valuable to show how missingness can be handled in trial-based CUAs and how different missingness approaches may influence findings. Furthermore, our findings align with the previous CEA, where there was no missingness on the effect outcome of *sickness absence days*, which also concluded that I-MORE compared with O-ACT was consistently not cost-effective in a healthcare perspective but consistently cost-effective in a limited societal perspective (15).

Another limitation is that we do not present an exhaustive overview of all methods for handling missingness. Hybrid approaches – for example, using a CCA for individuals with no HRQoL data and multiple imputation MI for those with partial data – may be more appropriate in certain contexts. However, our small sample size limited our ability to explore such methods, as excluding cases with no HRQoL data at any timepoint would have reduced the sample from 159 to 117. We chose to begin with simpler methods to demonstrate core concepts, while acknowledging that more tailored approaches may be preferable depending on the specific missingness pattern.

In conclusion, our findings, together with previous knowledge (15, 46), imply that I-MORE is cost-effective compared with O-ACT, when using HRQoL as the measure of health effect and when considering productivity loss, for individuals sick-listed with musculoskeletal and common mental disorders in Norway for a 24-month time horizon. While the overall cost-effectiveness conclusions were robust to the missing data method used, we demonstrated that using different missingness methods leads to variation in HRQoL and incremental QALYs. This highlights the importance of thoroughly analysing and appropriately addressing missingness in CUAs to ensure valid recommendations for new interventions. We advocate moving beyond a CCA as the default, and propose MI as the standard method for handling missing HRQoL data in a CUA. Further research is needed to guide the selection or integration of methods tailored to specific missing data contexts.

## Supplementary Material


