# Supplementary material for: A COST-UTILITY ANALYSIS FOR RETURN-TO-WORK INTERVENTIONS COMPARING ALTERNATIVE METHODS FOR HANDLING MISSING HEALTH-RELATED QUALITY OF LIFE DATA
Source: J Rehabil Med. 2025 Dec 1;57:42359. doi: 10.2340/jrm.v57.42359 (PMC12681026; doi:10.2340/jrm.v57.42359)
Supplement: Supplementary file 1 [file JRM-57-42359-s1.pdf]

Supplementary material has been published as submitted. It has not been copyedited, or typeset by Acta Dermato-Venereologica

Appendix S1: Additional Figures and Tables

Table SI. Cost per patient during 24 months of follow-up. Estimates are presented as means with standard deviation (SD). Costs in 2024 euros.

	O-ACT (n = 78)	I-MORE (n = 81)
	Mean (SD)	Mean (SD)
**Intervention program costs**	1,531	19,628
**Healthcare resource costs**	8,813 (14,071)	6,241 (10,051)
General practitioner[a]	1,474 (1,018)	1,446 (1,129)
Psychologist	1,92 (1,281)	105 (706)
Medical Imaging	328 (1,149)	190 (238)
Physiotherapist/chiropractor[b]	492 (1,118)	353 (709)
Private specialist	333 (1,383)	240 (938)
Psychiatric treatment		
Outpatient[c]	0 (0)	10 (92)
Inpatient	823 (2,607)	585 (1,916)
Rehabilitation	0 (0)	0 (0)
Outpatient	99 (641)	85 (766)
Inpatient	1,386 (9,494)	1,308 (9,041)
Somatic polyclinic		
Outpatient	1,287 (2,020)	789 (1,404)
Inpatient	2,398 (7,722)	1,130 (3,019)
**Total Costs: healthcare perspective**	10344 (14071)	25,869 (10,050)
**Costs of productivity loss**	134431 (85,459)	112,654 (86,595)
**Total Costs: including productivity loss**	147,174 (90,883)	144,525 (31,008)

O-ACT: outpatient acceptance and commitment therapy, *I-MORE*: inpatient multimodal occupational rehabilitation, [a] also includes emergency primary health care service and other physicians in primary care. [b] includes physiotherapist, manual physical therapist, psychomotor physiotherapy, and chiropractor. [c] includes substance abuse care

Table SII. Number and percentage of individuals with each pattern of missing data for HRQoL.

**Missingness pattern**				**O-ACT (n = 78)**	**I-MORE (n = 81)**	**Total (n = 159)**
U1	U5	U8	U14	n (%)	n (%)	n (%)
✓	✓	✓	✓	21 (27%)	21 (26%)	42 (26%)
✗	✗	✗	✗	26 (33%)	16 (20%)	42 (26%)
✓	✗	✗	✗	7 (9%)	13 (16%)	20 (13%)
✓	✓	✗	✗	8 (10%)	12 (15%)	20 (13%)
✓	✓	✓	✗	3 (4%)	7 (9%)	10 (6%)
✓	✓	✗	✓	3 (4%)	5 (6%)	8 (5%)
✓	✗	✓	✗	2 (3%)	2 (2%)	4 (3%)
✓	✗	✗	✓	2 (3%)	2 (2%)	4 (3%)
✓	✗	✓	✓	2 (3%)	2 (2%)	4 (3%)
✗	✗	✓	✗	3 (4%)	0 (0%)	3 (2%)
✗	✗	✓	✓	0 (0%)	1 (1%)	1 (<1%)
✗	✓	✗	✗	1 (1%)	0 (0%)	1 (<1%)

U1-14: health-related quality of life data at timepoints 1, 5, 8, 14, *O-ACT*: outpatient acceptance and commitment therapy, *I-MORE*: inpatient multimodal occupational rehabilitation, *Ticks (✓)*: observed data, *Crosses (✗)*: missing data

Table SIII. Health-related quality of life overtime by intervention using different methods to handing missing data. Estimates are presented as means after 5000 bootstrap simulations. HRQoL estimates using raw data are also shown for comparison.

Month from	Raw data		CCA (n=42)		SI (n=159)		MI (n=159)		LMM (n=159)	
baseline	Mean	(n)	Mean	(95% CB)	Mean	(95% CB)	Mean	(95% CB)	Mean	(95% CB)
**O-ACT**										
baseline			0.595		0.595		0.595		0.595	
1 month	0.578	(48)	0.556	(0.491, 0.621)	0.580	(0.534, 0.623)	0.580	(0.536, 0.624)	0.597	(0.555, 0.638)
5 months	0.611	(36)	0.597	(0.527, 0.667)	0.629	(0.576, 0.679)	0.624	(0.571, 0.674)	0.625	(0.577, 0.674)
8 months	0.627	(28)	0.587	(0.518, 0.656)	0.649	(0.586, 0.708)	0.642	(0.586, 0.696)	0.638	(0.583, 0.693)
14 months	0.658	(31)	0.606	(0.535, 0.682)	0.686	(0.619, 0.75)	0.654	(0.594, 0.721)	0.655	(0.585, 0.721)
15 months			0.612	(0.561, 0.664)	0.666	(0.617, 0.714)	0.656	(0.610, 0.704)	0.640	(0.588, 0.692)
24 months			0.612	(0.561, 0.664)	0.666	(0.617, 0.714)	0.656	(0.610, 0.704)	0.640	(0.588, 0.692)
**I-MORE**										
baseline			0.595		0.595		0.595		0.595	
1 month	0.607	(64)	0.581	(0.512, 0.646)	0.608	(0.572, 0.644)	0.609	(0.572, 0.644)	0.605	(0.571, 0.638)
5 months	0.660	(45)	0.615	(0.533, 0.69)	0.694	(0.644, 0.732)	0.682	(0.631, 0.731)	0.648	(0.607, 0.689)
8 months	0.657	(33)	0.639	(0.571, 0.709)	0.670	(0.621, 0.721)	0.663	(0.603, 0.718)	0.638	(0.591, 0.685)
14 months	0.646	(31)	0.618	(0.545, 0.689)	0.646	(0.584, 0.706)	0.658	(0.59, 0.726)	0.626	(0.568, 0.687)
15 months			0.612	(0.561, 0.664)	0.666	(0.617, 0.714)	0.656	(0.610, 0.704)	0.640	(0.588, 0.692)
24 months			0.612	(0.561, 0.664)	0.666	(0.617, 0.714)	0.656	(0.610, 0.704)	0.640	(0.588, 0.692)

CCA: complete case analysis, *SI:* single imputation, *MI*: multiple imputation, *LMM*: linear mixed models, *O-ACT*: outpatient acceptance and commitment therapy, *I-MORE*: inpatient multimodal occupational rehabilitation, *95% CB*: 95% confidence bound determined by taking the 2.5th percentile and the 97.5th percentile of the bootstrap replications

Fig. S1.1. Comparison of HRQoL overtime by intervention for the different methods of handing missing data after 5000 bootstrap simulations. Error bars represent 95% confidence bounds, determined by taking the 2.5th percentile and the 97.5th percentile of the bootstrap replications. Note: the scale on the HRQoL axis is condensed to 0.49-0.77 (instead of 0-1) for visibility. (a) Complete case analysis. (b) Single imputation. (c) Multiple imputation. (d) Linear mixed models. *O-ACT:* outpatient acceptance and commitment therapy, *I-MORE*: inpatient multimodal occupational rehabilitation.

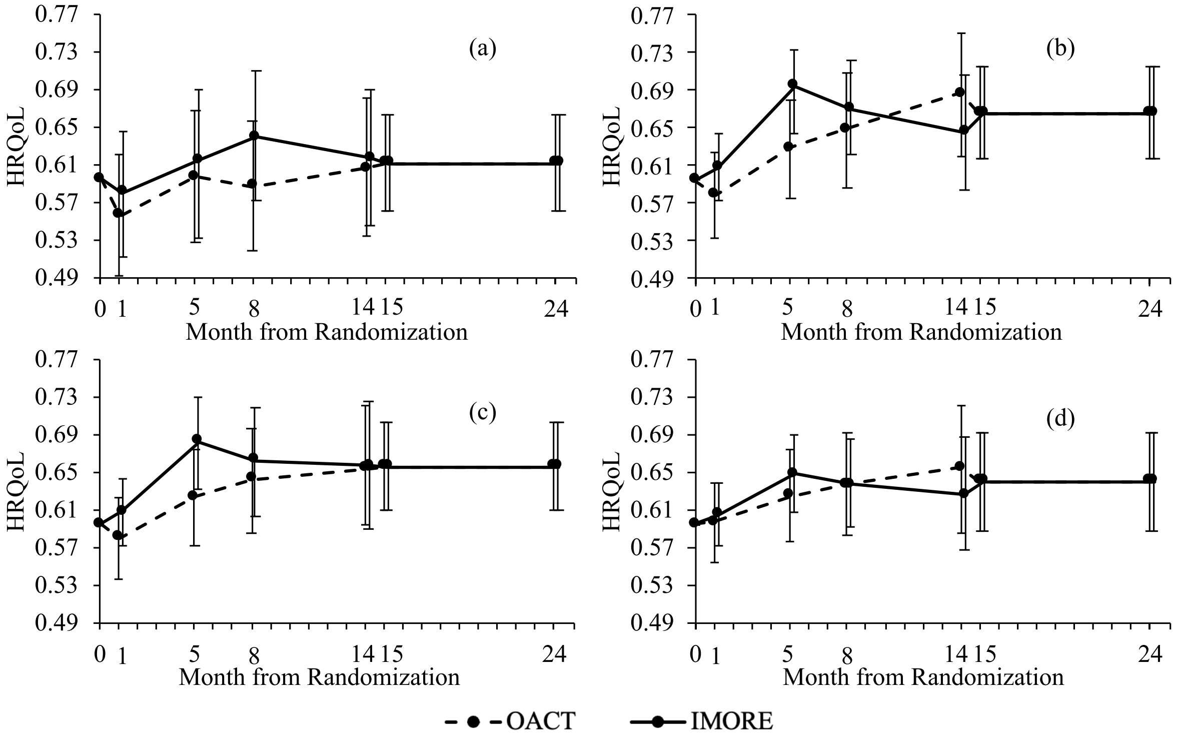

Fig. S1.2. Cost-effectiveness acceptability curves of each intervention using different methods of handling missing HRQoL data. (a) Complete case analysis. (b) Single imputation. (c) Multiple imputation. (d) Linear mixed models. *O-ACT*: outpatient acceptance and commitment therapy, *I-MORE*: inpatient multimodal occupational rehabilitation

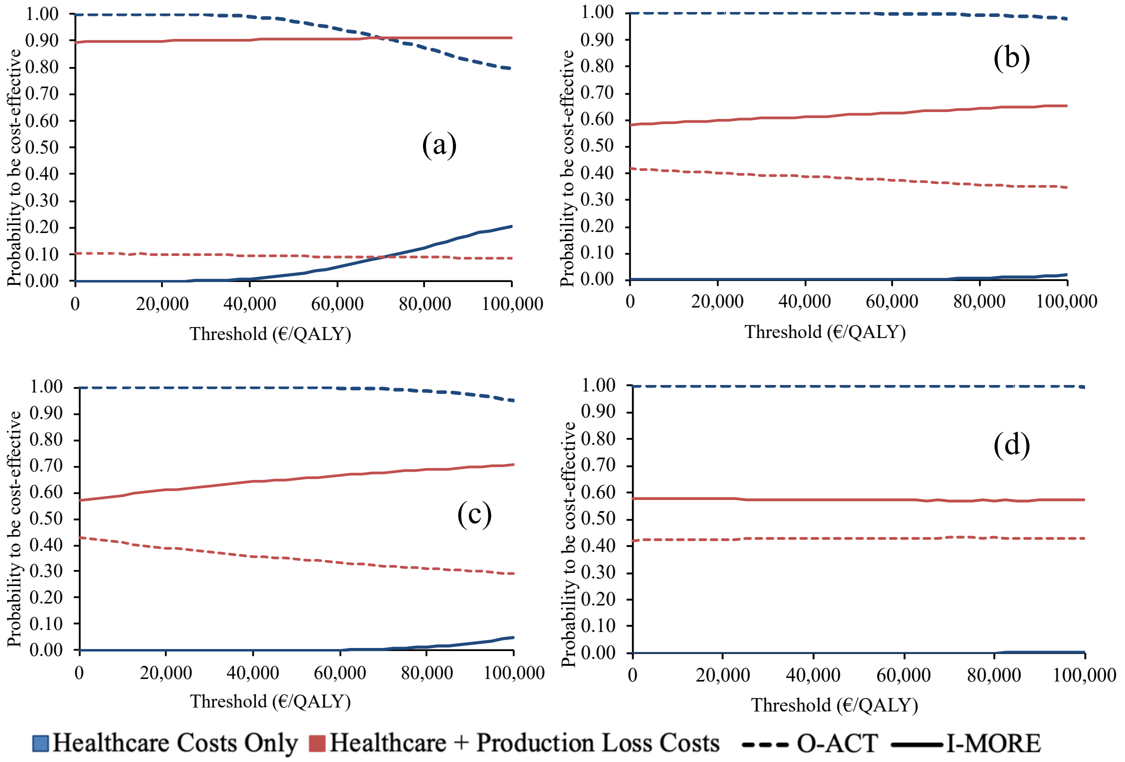

Table SIV. Cost-effectiveness results of MNAR analysis applying a 10% decrease and 10% increase to predicted HRQoL values. Estimates are presented as means after 5000 bootstrap simulations. Costs in Norwegian Krone. Missing HRQoL imputed using single imputation.

	Base-case	10% decrease	10% increase
QALYs O-ACT	1.300	1.225	1.373
QALYs I-MORE	1.319	1.248	1.388
Inc QALYs	0.019	0.023	0.015
**Healthcare perspective**			
Costs O-ACT	6,218	6,218	6,218
Costs I-MORE	15,577	15,577	15,577
Incremental costs	9,359	9,359	9,359
INMB[a]	-8,786	-8,658	-8,911
**Limited societal perspective**			
Costs O-ACT	71,785	71,785	71,785
Costs I-MORE	70,483	70,483	70,483
Incremental costs	-1,302	-1,302	-1,302
INMB[a]	1,875	2,003	1,750

MNAR: missing not at random, *HRQoL:* health-related quality of life, *O-ACT:* outpatient acceptance and commitment therapy, *I-MORE:* inpatient multimodal occupational rehabilitation, *QALY:* quality-adjusted life-year, *INMB*: incremental net monetary benefit.
[a]based on a cost-effectiveness threshold of €30,000 per QALY gained.

Table SV. Cost-effectiveness results of MNAR analysis applying a 10% decrease and 10% increase to predicted HRQoL values. Estimates are presented as means after 5000 bootstrap simulations. Costs in Norwegian Krone. Missing HRQoL imputed using multiple imputation.

	Base-case	10% decrease	10% increase
QALYs O-ACT	1.278	1.205	1.347
QALYs I-MORE	1.310	1.240	1.376
Inc QALYs	0.032	0.035	0.028
**Healthcare perspective**			
Costs O-ACT	6,240	6,240	6,240
Costs I-MORE	15,571	15,571	15,571
Incremental costs	9,331	9,331	9,331
INMB[a]	-8,380	-8,292	-8,479
**Limited societal perspective**			
Costs O-ACT	71,929	71,929	71,929
Costs I-MORE	70,565	70,565	70,565
Incremental costs	-1,365	-1,365	-1,365
INMB[a]	2,315	2,404	2,217

MNAR: missing not at random, *HRQoL:* health-related quality of life, *O-ACT:* outpatient acceptance and commitment therapy, *I-MORE:* inpatient multimodal occupational rehabilitation, *QALY:* quality-adjusted life-year, *INMB*: incremental net monetary benefit.
[a]based on a cost-effectiveness threshold of €30,000 per QALY gained.

Table SVI. Computational time to perform 5000 bootstraps for alternative methods to handle missing health-related quality of life data and obtain outcomes for a cost-utility analysis.

	Total Time	Number of Imputations	time/bootstrap
CCA	22s	0	-
SI	6m	5000	<0.1s
MI (m=10[a])	41h 10m	50,000	~30s
LMM	1h 44m	0	1.25s

CCA: complete case analysis, *SI*: single imputation, *MI*: multiple imputation, *LMM*: linear mixed models, *h*: hours, *m*: minutes, *s*: seconds. [a]number if imputed sets is 10.

Appendix S2: Linear Mixed Model

LMM predicts values using maximum likelihood estimation. The term “mixed model” comes from the combination of fixed effects and random effects. Including a random effect allows for different individuals to have different trends in HRQoL overtime, rather than assuming the same trend for all individuals (19). Our model included two variables in addition to the chosen covariates: time and an interaction between time and intervention to capture the treatment effect of I-MORE compared to O-ACT at each timepoint. A simplified version of the LMM model, without covariates, is represented as:

$$HRQOL_{it} = \beta_{0i} + \beta_{1i}TIME + \beta_{2i}TIME_jINT_i + \beta_{3i}INT_i + \omega_i + \epsilon_{it} \qquad (1)$$

where $HRQOL_{it}$ represents HRQoL for individual i ($i = 1,\ldots,159$) at timepoint t ($t = 0,1,5,8,14,15,24$). $TIME_j$ and INT_i represent the timepoint that HRQoL was measured and the intervention, respectively. $TIME_jINT_i$ is the interaction between time and intervention at each timepoint. The coefficients β_{0-3} represent the model parameters. β_2, specifically, represents the treatment effect of I-MORE compared to O-ACT at each timepoint. ω_i represents the random intercept, which accounts for the variation in outcome between individuals. ϵ_{it} is the error term for each individual at each timepoint.

Apendix S3: Stata Code

Impute baseline HRQoL and other variables

```
*Impute baseline HRQoL using mean of 15D at month 1
sum score15D1m
replace score15D0 = r(mean)

*Impute baseline variables using median
sum education, detail
replace education = r(p50) if missing(education0)

sum disability0, detail
replace disability0 = r(p50) if missing(disability0)

sum strong_pain0, detail
replace strong_pain0 = r(p50) if missing(strong_pain0)

sum avg_pain0, detail
replace avg_pain0 = r(p50) if missing(avg_pain0)

sum hadsa0, detail
replace hadsa0 = r(p50) if missing(hadsa0)

sum hadsd0, detail
replace hadsd0 = r(p50) if missing(hadsd0)
```

CCA

Obtaining CUA outcomes and HRQoL values at each timepoint using a CCA for 5000 bootstrap simulations. Including imputation of HRQoL at the 24-month timepoint

```
*drop all individuals with missing HRQoL data
drop if missing(score15D1m) | missing(score15D5m) | missing(score15D8m) |
missing(score15D14m) //drop all observations with missing data


program define myboot_CCA, rclass
            preserve //restores the data to before the program ran
            bsample, strata(intervention) //bootstrap sample based on users
and keeping same individuals in each intervention

            *=======calculate QALYs using area under the curve
            *15 month imputed with mean 14 month and maintained till 24
month
            sum score15D14m
            replace score15D15m = r(mean)
            replace score15D24m = r(mean)

            generate QALYs = (score15D0 + score15D1m)/2*(1/12) +
(score15D1m+score15D5m)/2*(4/12) + (score15D5m+score15D8m)/2*(3/12) +
(score15D8m+score15D14m)/2*(6/12) + (score15D14m + score15D15m)/2*(1/12) +
(score15D15m + score15D24m)/2*(9/12)

            *=======return all outputs by intervention
            *=======FOR EXAMPLE=======

            *QALYs
```

```
            sum QALYs if intervention == 0 //OACT
            return scalar QALYs_OACT = r(mean)
            sum QALYs if intervention == 1 //IMORE
            return scalar QALYs_IMORE = r(mean)

      end

*===========perform program for 5000 bootstrapped samples
      simulate QALYs_OACT = r(QALYs_OACT) totalCostHC_24m_OACT =
r(totalCostHC_24m_OACT) totalCostPL_24m_OACT = r(totalCostPL_24m_OACT)
score15D1m_OACT = r(score15D1m_OACT) score15D5m_OACT = r(score15D5m_OACT)
score15D8m_OACT = r(score15D8m_OACT) score15D14m_OACT = r(score15D14m_OACT)
score15D15m_OACT = r(score15D15m_OACT) QALYs_IMORE = r(QALYs_IMORE)
totalCostHC_24m_IMORE = r(totalCostHC_24m_IMORE) totalCostPL_24m_IMORE =
r(totalCostPL_24m_IMORE) score15D1m_IMORE = r(score15D1m_IMORE)
score15D5m_IMORE = r(score15D5m_IMORE) score15D8m_IMORE =
r(score15D8m_IMORE) score15D14m_IMORE = r(score15D14m_IMORE)
score15D15m_IMORE = r(score15D15m_IMORE), ///
      reps(5000) seed(81298) saving("working directory", replace):
myboot_CCA
```

SI

Imputation of missing HRQoL values using SI and obtaining CUA outcomes and HRQoL values at each timepoint for 5000 bootstrap simulations. Including the MNAR sensitivity analysis and scenario analyses and imputation of HRQoL at the 24-month timepoint.

```
program define myboot_SI, rclass
            preserve //restores the data to before the program ran
            bsample, strata(intervention) //bootstrap sample based on users
and keeping same individuals in each intervention

            *1month
            regress score15D1m age i.gender i.marital i.diagnosis hadsa0
hadsd0 i.intervention absence0_1 absence1
            predict score15D1m_p,xb //get predicted values

            replace score15D1m_base = score15D1m_p       if
missing(score15D1m)
            replace score15D1m_dec = score15D1m_p * 0.9 if
missing(score15D1m)
            replace score15D1m_inc = score15D1m_p * 1.1 if
missing(score15D1m)

            foreach var of varlist score15D1m_base score15D1m_dec
score15D1m_inc {
                  replace `var' = 1 if `var' > 1 //if above 1
            }

            *5 months
            regress score15D5m score15D1m_base age i.gender i.marital
i.diagnosis hadsa0 hadsd0 i.intervention absence0_5 absence5
            predict score15D5m_p,xb //get predicted values

            replace score15D5m_base = score15D5m_p       if
missing(score15D5m)
```

```
            replace score15D5m_dec = score15D5m_p * 0.9 if
missing(score15D5m)
            replace score15D5m_inc = score15D5m_p * 1.1 if
missing(score15D5m)

            foreach var of varlist score15D5m_base score15D5m_dec
score15D5m_inc {
                  replace `var' = 1 if `var' >1 //if above 1
            }

            *8 months
            regress score15D8m score15D5m_base age i.gender i.marital
i.diagnosis hadsa0 hadsd0 i.intervention absence0_8 absence8
            predict score15D8m_p,xb //get predicted values
            replace score15D8m_base = score15D8m_p       if
missing(score15D8m)
            replace score15D8m_dec = score15D8m_p * 0.9 if
missing(score15D8m)
            replace score15D8m_inc = score15D8m_p * 1.1 if
missing(score15D8m)

            foreach var of varlist score15D8m_base score15D8m_dec
score15D8m_inc {
                  replace `var' = 1 if `var' >1 //if above 1
            }

            *14 months
            regress score15D14m score15D8m_base age i.gender i.marital
i.diagnosis hadsa0 hadsd0 i.intervention absence0_14 absence14
            predict score15D14m_p,xb //get predicted values
            replace score15D14m_base = score15D14m_p       if
missing(score15D14m)
            replace score15D14m_dec = score15D14m_p * 0.9 if
missing(score15D14m)
            replace score15D14m_inc = score15D14m_p * 1.1 if
missing(score15D14m)

            foreach var of varlist score15D14m_base score15D14m_dec
score15D14m_inc {
                  replace `var' = 1 if `var' >1 //if above 1
            }

            *=======calculate QALYs for base case and MNAR

            *15 month imputed with mean 14 month and maintained till 24
month


            local list_15D_imp _base _dec _inc
            foreach var of local list_15D_imp {

                  *first impute 15m and 24m using average of 14m
                  sum score15D14m`var'
                  gen score15D15m`var' = r(mean)
                  gen score15D24m`var' = r(mean)

                  gen QALYs`var' = (score15D0 + score15D1m`var')/2*(1/12) +
(score15D1m`var' + score15D5m`var')/2*(4/12) + (score15D5m`var' +
score15D8m`var')/2*(3/12) + (score15D8m`var' + score15D14m`var')/2*(6/12) +
(score15D14m`var' + score15D15m`var')/2*(1/12) + (score15D15m`var' +
score15D24m`var')/2*(9/12)
```

```
            }

            *=======calculate QALYs for 24m scenario analyses

            *Scenario 1: 24 month imputed with mean 14 month
            sum score15D14m_base
            gen score15D24m_S1 = r(mean)

            *Scenario 2: 24 month imputed with LVCF
            gen score15D24m_S2 = score15D14m_base

            local list_24m_base score15D24m_S1 score15D24m_S2
            local i = 1
            foreach var of local list_24m_base {
                  gen QALYs_`i' = (score15D0 + score15D1m_base)/2*(1/12) +
(score15D1m_base + score15D5m_base)/2*(4/12) + (score15D5m_base +
score15D8m_base)/2*(3/12) + (score15D8m_base + score15D14m_base)/2*(6/12) +
(score15D14m_base + `var')/2*(10/12)
                  local i = `i' + 1
            }

            *=======return all outputs by intervention

            *=======FOR EXAMPLE=======
            *QALYs
            sum QALYs_base if intervention == 0 //OACT
            return scalar QALYs_base_OACT = r(mean)
            sum QALYs_base if intervention == 1 //IMORE
            return scalar QALYs_base_IMORE = r(mean)


      end

*===========perform program for 5000 bootstrapped samples

      simulate QALYs_base_OACT = r(QALYs_base_OACT) QALYs_dec_OACT =
r(QALYs_dec_OACT) QALYs_inc_OACT = r(QALYs_inc_OACT) QALYs_1_OACT =
r(QALYs_1_OACT) QALYs_2_OACT = r(QALYs_2_OACT) QALYs_14m_OACT =
r(QALYs_14m_OACT) totalCostHC_24m_OACT = r(totalCostHC_24m_OACT)
totalCostPL_24m_OACT = r(totalCostPL_24m_OACT) totalCostHC_14m_OACT =
r(totalCostHC_14m_OACT) totalCostPL_14m_OACT = r(totalCostPL_14m_OACT)
score15D1m_base_OACT = r(score15D1m_base_OACT) score15D1m_dec_OACT =
r(score15D1m_dec_OACT) score15D1m_inc_OACT = r(score15D1m_inc_OACT)
score15D5m_base_OACT = r(score15D5m_base_OACT) score15D5m_dec_OACT =
r(score15D5m_dec_OACT) score15D5m_inc_OACT = r(score15D5m_inc_OACT)
score15D8m_base_OACT = r(score15D8m_base_OACT) score15D8m_dec_OACT =
r(score15D8m_dec_OACT) score15D8m_inc_OACT = r(score15D8m_inc_OACT)
score15D14m_base_OACT = r(score15D14m_base_OACT) score15D14m_dec_OACT =
r(score15D14m_dec_OACT) score15D14m_inc_OACT = r(score15D14m_inc_OACT)
score15D15m_base_OACT = r(score15D15m_base_OACT) score15D15m_dec_OACT =
r(score15D15m_dec_OACT) score15D15m_inc_OACT = r(score15D15m_inc_OACT)
score15D24m_base_OACT = r(score15D24m_base_OACT) score15D24m_dec_OACT =
r(score15D24m_dec_OACT) score15D24m_inc_OACT = r(score15D24m_inc_OACT)
score15D24m_S1_OACT = r(score15D24m_S1_OACT) score15D24m_S2_OACT =
r(score15D24m_S2_OACT) QALYs_base_IMORE = r(QALYs_base_IMORE)
QALYs_dec_IMORE = r(QALYs_dec_IMORE) QALYs_inc_IMORE = r(QALYs_inc_IMORE)
QALYs_1_IMORE = r(QALYs_1_IMORE) QALYs_2_IMORE = r(QALYs_2_IMORE)
QALYs_14m_IMORE = r(QALYs_14m_IMORE) totalCostHC_24m_IMORE =
r(totalCostHC_24m_IMORE) totalCostPL_24m_IMORE = r(totalCostPL_24m_IMORE)
totalCostHC_14m_IMORE = r(totalCostHC_14m_IMORE) totalCostPL_14m_IMORE =
r(totalCostPL_14m_IMORE) score15D1m_base_IMORE = r(score15D1m_base_IMORE)
```

```
score15D1m_dec_IMORE = r(score15D1m_dec_IMORE) score15D1m_inc_IMORE =
r(score15D1m_inc_IMORE) score15D5m_base_IMORE = r(score15D5m_base_IMORE)
score15D5m_dec_IMORE = r(score15D5m_dec_IMORE) score15D5m_inc_IMORE =
r(score15D5m_inc_IMORE) score15D8m_base_IMORE = r(score15D8m_base_IMORE)
score15D8m_dec_IMORE = r(score15D8m_dec_IMORE) score15D8m_inc_IMORE =
r(score15D8m_inc_IMORE) score15D14m_base_IMORE = r(score15D14m_base_IMORE)
score15D14m_dec_IMORE = r(score15D14m_dec_IMORE) score15D14m_inc_IMORE =
r(score15D14m_inc_IMORE) score15D15m_base_IMORE = r(score15D15m_base_IMORE)
score15D15m_dec_IMORE = r(score15D15m_dec_IMORE) score15D15m_inc_IMORE =
r(score15D15m_inc_IMORE) score15D24m_base_IMORE = r(score15D24m_base_IMORE)
score15D24m_dec_IMORE = r(score15D24m_dec_IMORE) score15D24m_inc_IMORE =
r(score15D24m_inc_IMORE) score15D24m_S1_IMORE = r(score15D24m_S1_IMORE)
score15D24m_S2_IMORE = r(score15D24m_S2_IMORE), ///
      reps(5000) seed(81298) saving("working directory", replace):
myboot_SI
```

MI

Imputation of missing HRQoL values using MI and obtaining CUA outcomes and HRQoL values at each timepoint for 5000 bootstrap simulations. Including the MNAR sensitivity analysis and scenario analyses and imputation of HRQoL at the 24-month timepoint.

```
program define myboot_MI, rclass
            preserve //restores the data to before the program ran
            bsample, strata(intervention) //bootstrap sample keeps same
number of individuals in each intervention

            mi set flong //set data to be stored in longform
            mi register imputed score15D1m_base score15D5m_base
score15D8m_base score15D14m_base //variables to be imputed

            mi impute chained (pmm, knn(5)) score15D1m_base score15D5m_base
score15D8m_base score15D14m_base = age i.gender i.marital i.diagnosis
hadsa0 hadsd0 absence0_24, add(10) force by (intervention) //multiple
imputate by intervention

            *=======Create variables for MNAR adjusting imputed values
            local list_score15D score15D1m score15D5m score15D8m
score15D14m
            foreach var of local list_score15D{
                  replace `var'_dec = `var'_base * 0.9 if
missing(`var'_dec)
                  replace `var'_inc = `var'_base * 1.1 if
missing(`var'_inc)
                  replace `var'_inc = 1 if `var'_inc > 1
            }

            *=======calculate QALYs for base case and MNAR
            *15 month imputed with mean 14 month and maintained till 24
month

            local list_15D_imp _base _dec _inc
            foreach var of local list_15D_imp {
                  mi estimate: mean score15D14m`var' //24month follow-up
                  mi passive: gen score15D15m`var' = el(e(b_mi),1,1)
                  mi passive: gen score15D24m`var' = el(e(b_mi),1,1)
```

```
                mi passive: gen QALYs`var' = (score15D0 +
score15D1m`var')/2*(1/12) + (score15D1m`var' + score15D5m`var')/2*(4/12) +
(score15D5m`var' + score15D8m`var')/2*(3/12) + (score15D8m`var' +
score15D14m`var')/2*(6/12) + (score15D14m`var' + score15D15m`var')/2*(1/12)
+ (score15D15m`var' + score15D24m`var')/2*(9/12) //gen QALys for all m
imputed sets'
            }

            *=======calculate QALYs for 24m scenario analyses
            *Scenario 1: 24 month imputed with mean 14 month
            mi estimate: mean score15D14m_base //24month follow-up
            mi passive: gen score15D24m_S1 = el(e(b_mi),1,1)

            *Scenario 2: 24 month imputed with LVCF
            mi passive: gen score15D24m_S2 = score15D14m_base

            local list_24m_base score15D24m_S1 score15D24m_S2
            local i = 1
            foreach var of local list_24m_base {
                mi passive: gen QALYs_`i' = (score15D0 +
score15D1m_base)/2*(1/12) + (score15D1m_base + score15D5m_base)/2*(4/12) +
(score15D5m_base + score15D8m_base)/2*(3/12) + (score15D8m_base +
score15D14m_base)/2*(6/12) + (score15D14m_base + `var')/2*(10/12)
                local i = `i' + 1
            }

            *=======calculate QALYs only until first year followup to
remain consistent with CCA
            mi passive: generate QALYs_14m = (score15D0 +
score15D1m_base)/2*(1/12) + (score15D1m_base + score15D5m_base)/2*(4/12) +
(score15D5m_base + score15D8m_base)/2*(3/12) + (score15D8m_base +
score15D14m_base)/2*(6/12)

            *QALYs
            *=======return all outputs by intervention

            *=======FOR EXAMPLE=======
            mi estimate: mean QALYs_base if intervention == 0 //OACT
            return scalar QALYs_base_OACT = el(e(b_mi),1,1)
            mi estimate: mean QALYs_base if intervention == 1 //IMORE
            return scalar QALYs_base_IMORE = el(e(b_mi),1,1)
end

*===========perform program for 5000 bootstrapped samples

      simulate QALYs_base_OACT = r(QALYs_base_OACT) QALYs_dec_OACT =
r(QALYs_dec_OACT) QALYs_inc_OACT = r(QALYs_inc_OACT) QALYs_1_OACT =
r(QALYs_1_OACT) QALYs_2_OACT = r(QALYs_2_OACT) QALYs_14m_OACT =
r(QALYs_14m_OACT) totalCostHC_24m_OACT = r(totalCostHC_24m_OACT)
totalCostPL_24m_OACT = r(totalCostPL_24m_OACT) totalCostHC_14m_OACT =
r(totalCostHC_14m_OACT) totalCostPL_14m_OACT = r(totalCostPL_14m_OACT)
score15D1m_base_OACT = r(score15D1m_base_OACT) score15D1m_dec_OACT =
r(score15D1m_dec_OACT) score15D1m_inc_OACT = r(score15D1m_inc_OACT)
score15D5m_base_OACT = r(score15D5m_base_OACT) score15D5m_dec_OACT =
r(score15D5m_dec_OACT) score15D5m_inc_OACT = r(score15D5m_inc_OACT)
score15D8m_base_OACT = r(score15D8m_base_OACT) score15D8m_dec_OACT =
r(score15D8m_dec_OACT) score15D8m_inc_OACT = r(score15D8m_inc_OACT)
score15D14m_base_OACT = r(score15D14m_base_OACT) score15D14m_dec_OACT =
r(score15D14m_dec_OACT) score15D14m_inc_OACT = r(score15D14m_inc_OACT)
score15D15m_base_OACT = r(score15D15m_base_OACT) score15D15m_dec_OACT =
r(score15D15m_dec_OACT) score15D15m_inc_OACT = r(score15D15m_inc_OACT)
```

```
score15D24m_base_OACT = r(score15D24m_base_OACT) score15D24m_dec_OACT =
r(score15D24m_dec_OACT) score15D24m_inc_OACT = r(score15D24m_inc_OACT)
score15D24m_S1_OACT = r(score15D24m_S1_OACT) score15D24m_S2_OACT =
r(score15D24m_S2_OACT) QALYs_base_IMORE = r(QALYs_base_IMORE)
QALYs_dec_IMORE = r(QALYs_dec_IMORE) QALYs_inc_IMORE = r(QALYs_inc_IMORE)
QALYs_1_IMORE = r(QALYs_1_IMORE) QALYs_2_IMORE = r(QALYs_2_IMORE)
QALYs_14m_IMORE = r(QALYs_14m_IMORE) totalCostHC_24m_IMORE =
r(totalCostHC_24m_IMORE) totalCostPL_24m_IMORE = r(totalCostPL_24m_IMORE)
totalCostHC_14m_IMORE = r(totalCostHC_14m_IMORE) totalCostPL_14m_IMORE =
r(totalCostPL_14m_IMORE) score15D1m_base_IMORE = r(score15D1m_base_IMORE)
score15D1m_dec_IMORE = r(score15D1m_dec_IMORE) score15D1m_inc_IMORE =
r(score15D1m_inc_IMORE) score15D5m_base_IMORE = r(score15D5m_base_IMORE)
score15D5m_dec_IMORE = r(score15D5m_dec_IMORE) score15D5m_inc_IMORE =
r(score15D5m_inc_IMORE) score15D8m_base_IMORE = r(score15D8m_base_IMORE)
score15D8m_dec_IMORE = r(score15D8m_dec_IMORE) score15D8m_inc_IMORE =
r(score15D8m_inc_IMORE) score15D14m_base_IMORE = r(score15D14m_base_IMORE)
score15D14m_dec_IMORE = r(score15D14m_dec_IMORE) score15D14m_inc_IMORE =
r(score15D14m_inc_IMORE) score15D15m_base_IMORE = r(score15D15m_base_IMORE)
score15D15m_dec_IMORE = r(score15D15m_dec_IMORE) score15D15m_inc_IMORE =
r(score15D15m_inc_IMORE) score15D24m_base_IMORE = r(score15D24m_base_IMORE)
score15D24m_dec_IMORE = r(score15D24m_dec_IMORE) score15D24m_inc_IMORE =
r(score15D24m_inc_IMORE) score15D24m_S1_IMORE = r(score15D24m_S1_IMORE)
score15D24m_S2_IMORE = r(score15D24m_S2_IMORE), ///
      reps(330) seed(33130) saving("/Volumes/p806/Cindy/bootstrap_MI2.dta",
replace): myboot_MI
```

LMM

Estimating HRQoL values at each timepoint using LMM and obtaining CUA outcomes and HRQoL values at each timepoint for 5000 bootstrap simulations. Including imputation of HRQoL at the 24-month timepoint.

```
*Prepare data to transform to long form
rename (score15D1m score15D5m score15D8m score15D14m score15D24m)
(score15D1 score15D5 score15D8 score15D14 score15D24) //rename 15d
variables

rename (absence0_1 absence0_5 absence0_8 absence0_14) (cum_abs1 cum_abs5
cum_abs8 cum_abs14) //rename absence variables

gen cum_abs0 = absence0

*keep only relevant data to the analysis
keep score15D0 score15D1 score15D5 score15D8 score15D14 user age gender
marital diagnosis hadsa0 hadsd0 intervention totalCostPL_24m
totalCostHC_24m totalCostPL_14m totalCostHC_14m absence0 absence1 absence5
absence8 absence14 cum_abs0 cum_abs1 cum_abs5 cum_abs8 cum_abs14

*reshape data to long
reshape long score15D absence cum_abs, i(user) j(month)
label drop tidspkt
label variable cum_abs "cumulative absence up to month"
label variable absence "absence in that month"

*create new variable for timepoint (to be used in bootstrap)
gen timepoint = month
replace timepoint = 2 if timepoint==5
replace timepoint = 3 if timepoint==8
replace timepoint = 4 if timepoint==14
```

```
program define myboot_LMM, rclass
            preserve //restores the data to before the program ran
            bsample, cluster(user) strata(intervention) //bootstrap sample
based on users and keeping same individuals in each intervention

            *create new user id since boostrap sample will mess it up

            sort month user
            gen id=_n
            replace id = id-159*timepoint
            sort id month
            replace user = id
            drop id

            mixed score15D i.month i.month#i.intervention age i.gender
i.marital i.diagnosis hadsa0 hadsd0 i.intervention absence cum_abs || user:
//, residuals(unstructured, t(month))

            *estimates for Utility per arm at each time-point
            margins i.month#i.intervention
            *display el(r(b),1,10 //access each estimate

            *=======calculate QALYs for base case
            return scalar QALYs_OACT = (el(r(b),1,1) +
el(r(b),1,3))/2*(1/12) + (el(r(b),1,3) + el(r(b),1,5))/2*(4/12) +
(el(r(b),1,5) + el(r(b),1,7))/2*(3/12) + (el(r(b),1,7) +
el(r(b),1,9))/2*(6/12) + (el(r(b),1,9) +
(el(r(b),1,9)+el(r(b),1,10))/2)/2*(1/12) + ((el(r(b),1,9)+el(r(b),1,10))/2
+ (el(r(b),1,9)+el(r(b),1,10))/2)/2*(9/12)

            return scalar QALYs_IMORE = (el(r(b),1,2) +
el(r(b),1,4))/2*(1/12) + (el(r(b),1,4) + el(r(b),1,6))/2*(4/12) +
(el(r(b),1,6) + el(r(b),1,8))/2*(3/12) + (el(r(b),1,8) +
el(r(b),1,10))/2*(6/12) + (el(r(b),1,10) +
(el(r(b),1,9)+el(r(b),1,10))/2)/2*(1/12) + ((el(r(b),1,9)+el(r(b),1,10))/2
+ (el(r(b),1,9)+el(r(b),1,10))/2)/2*(9/12)


            *=======calculate QALYs for 24m scenario analyses
            *Scenario 1: 24 month imputed with mean 14 month
            return scalar QALYs_1_OACT = (el(r(b),1,1) +
el(r(b),1,3))/2*(1/12) + (el(r(b),1,3) + el(r(b),1,5))/2*(4/12) +
(el(r(b),1,5) + el(r(b),1,7))/2*(3/12) + (el(r(b),1,7) +
el(r(b),1,9))/2*(6/12) + (el(r(b),1,9) +
(el(r(b),1,9)+el(r(b),1,10))/2)/2*(10/12)

            return scalar QALYs_1_IMORE = (el(r(b),1,2) +
el(r(b),1,4))/2*(1/12) + (el(r(b),1,4) + el(r(b),1,6))/2*(4/12) +
(el(r(b),1,6) + el(r(b),1,8))/2*(3/12) + (el(r(b),1,8) +
el(r(b),1,10))/2*(6/12) + (el(r(b),1,10) +
(el(r(b),1,9)+el(r(b),1,10))/2)/2*(10/12)

            *Scenario 2: 24 month imputed with LVCF
            return scalar QALYs_2_OACT = (el(r(b),1,1) +
el(r(b),1,3))/2*(1/12) + (el(r(b),1,3) + el(r(b),1,5))/2*(4/12) +
(el(r(b),1,5) + el(r(b),1,7))/2*(3/12) + (el(r(b),1,7) +
el(r(b),1,9))/2*(6/12) + (el(r(b),1,9) + el(r(b),1,9))/2*(10/12)

            return scalar QALYs_2_IMORE = (el(r(b),1,2) +
el(r(b),1,4))/2*(1/12) + (el(r(b),1,4) + el(r(b),1,6))/2*(4/12) +
```

```
(el(r(b),1,6) + el(r(b),1,8))/2*(3/12) + (el(r(b),1,8) +
el(r(b),1,10))/2*(6/12) + (el(r(b),1,10) + el(r(b),1,10))/2*(10/12)

            *=======calculate QALYs only until first year followup to
remain consistent with CCA
            return scalar QALYs_14m_OACT = (el(r(b),1,1) +
el(r(b),1,3))/2*(1/12) + (el(r(b),1,3) + el(r(b),1,5))/2*(4/12) +
(el(r(b),1,5) + el(r(b),1,7))/2*(3/12) + (el(r(b),1,7) +
el(r(b),1,9))/2*(6/12)

            return scalar QALYs_14m_IMORE = (el(r(b),1,2) +
el(r(b),1,4))/2*(1/12) + (el(r(b),1,4) + el(r(b),1,6))/2*(4/12) +
(el(r(b),1,6) + el(r(b),1,8))/2*(3/12) + (el(r(b),1,8) +
el(r(b),1,10))/2*(6/12)

            *=======return all other outputs by intervention

            *=======FOR EXAMPLE=======
            *15D scores at each timepoint
            return scalar score15D1m_OACT = el(r(b),1,3)
            return scalar score15D1m_IMORE = el(r(b),1,4)
end

*===========perform program for 5000 bootstrapped samples

simulate lmm_QALYs_OACT=r(lmm_QALYs_OACT)
lmm_QALYs_IMORE=r(lmm_QALYs_IMORE)
lmm_totalCostHC24m_OACT=r(lmm_totalCostHC24m_OACT)
lmm_totalCostHC24m_IMORE=r(lmm_totalCostHC24m_IMORE)
lmm_totalCostPL24m_OACT=r(lmm_totalCostPL24m_OACT)
lmm_totalCostPL24m_IMORE=r(lmm_totalCostPL24m_IMORE)
lmm_score15D1m_OACT=r(lmm_score15D1m_OACT)
lmm_score15D1m_IMORE=r(lmm_score15D1m_IMORE)
lmm_score15D5m_OACT=r(lmm_score15D5m_OACT)
lmm_score15D5m_IMORE=r(lmm_score15D5m_IMORE)
lmm_score15D8m_OACT=r(lmm_score15D8m_OACT)
lmm_score15D8m_IMORE=r(lmm_score15D8m_IMORE)
lmm_score15D14m_OACT=r(lmm_score15D14m_OACT)
lmm_score15D14m_IMORE=r(lmm_score15D14m_IMORE)lmm_score15D24m_OACT=r(lmm_sc
ore15D24m_OACT) lmm_score15D24m_IMORE=r(lmm_score15D24m_IMORE), ///
      reps(5000) seed(81298)
saving("/Volumes/p806/Cindy/bootstrap_LMMfinal.dta", replace): myboot_LMM
      //reps(5000): perform 5000 simulations
```

Appendix S4: Choosing explanatory variables / covariates

Variable	Description	Values in Stata	Variable name in Stata
Age	Age at program start/baseline	Continuous variable (>0)	age
Gender		0 = female, 1 = male	gender
Education	Education at baseline	0 = lower education, 1 = higher education	education
Marital Status	Marital status at baseline	0 = single, 1 = unmarried, 2 = married, 3 = cohabitant, 4 = widow/widower, 5 = divorced, 7 = separated	marital
Disability Status	Disability status at baseline	0 = no disability, 1 = disability	disability
BPI: Strong pain	strongest pain experienced in the last week (measured at baseline and each follow-up)	Integer values from 1 to 10	strong_pain0, strong_pain1m, strong_pain5m, strong_pain8m, strong_pain14m
BPI: Average pain	Average pain experienced in the last week (measured at baseline and each follow-up)	Integer values from 1 to 10	avg_pain0, avg_pain1m, avg_pain5m, avg_pain8m, avg_pain14m
HADS: Depression	Hospital Anxiety and Depression Scale: Depression (measured at baseline and each follow-up)	Range: 0-21	hadsd0, hadsd1m, hadsd5m, hadsd8m, hadsd14m
HADS: Anxiety	Hospital Anxiety and Depression Scale: Anxiety (measured at baseline and each follow-up)	Range: 0-21	hadsa0, hadsa1m, hadsa5m, hadsa8m, hadsa14m
ICPC-2 Diagnosis	International Classification of Primary Care Diagnosis	1 = "A: general and unspecified", 2 = L: musculoskeletal, 3 = P: psychiatric	

Intervention group	Randomized treatment group	0 = O-ACT, 1 = I-MORE	
Absence in each month	Days on sick leave per month	Integer values from 1 to 31	absence1, absence5, absence8, absence14, absence24
Cumulative Absence	Cumulative days on sick leave up to the timepoint	Positive integers	absence0_1, absence0_5, absence0_8, absence0_14, absence0_24
Previous HRQoL	The HRQoL value / 15D score at the previous timepoint	Continuous variable, range (-0.52 to 1)	score15D1m, score15D5m, score15D8m, score15D14m

Table SVII. Logistic regression for missingness of health-related quality of life on baseline variables, only statistically significant variables shown.

	Odds ratio	95% Confidence Interval
**1 month**		
Age	0.92**	(0.86, 0.99)
Gender (male)	2.97*	(0.91, 9.63)
Depression[a]	1.12**	(0.70, 1.00)
Absence up to 1 month	1.09*	(0.98, 1.20)
**5 Months**		
Age	0.80***	(0.71, 0.89)
Married[b]	7.37*	(0.75, 72.8)
**8 months**		
Missingness at 5 months	25.26***	(7.14, 89.36)
Cohabitant	0.21*	(0.04, 1.07)
Absence in month 8	1.08*	(1.00, 1.18)
Absence up to 8 months	0.99**	(0.97, 1.00)
**14 months**		
Missingness at 8 months	1765.05***	(73.21, 42555.15)
Age	0.84***	(0.76, 0.94)
Married[b]	12.72**	(1.02, 159.29)
Cohabitant[b]	29.81**	(1.50, 591.30)
Anxiety[a]	1.35**	(1.03, 1.76)
Depression[a]	0.70***	(0.54, 0.91)
I-MORE	6.78**	(1.48, 31.02)

I-MORE: inpatient multimodal occupational rehabilitation. [a] measured using Hospital Anxiety and Depression Scale. [b] reference group: single. * $p < 0.1$. ** $p < 0.05$. *** $p < 0.01$

Table SVIII. Logistic regression for missingness of HRQoL on previous observed HRQoL value

	Odds ratio	95% Confidence Interval
**1 month**	-	-
**5 months**		
HRQoL at 1 month	38.74**	(1.91, 785.19)
**8 months**		
HRQoL at 1 month	0.13	(0.00, 14.24)
HRQoL at 5 months	42.14*	(0.50, 3531.44)
**14 months**		
HRQoL at 1 month	0.00**	(2.27e-08, 0.64)
HRQoL at 5 months	41.18	(0.02, 80433.69)
HRQoL at 8 months	1619.30**	(1.16, 2260018)

HRQoL: health-related quality of life. * p < 0.1. ** p < 0.05. *** p < 0.01

Table SIX. Linear regression of HRQoL on baseline variables and previous HRQoL, only statistically significant variables shown

	Coefficient	Standard Error	Adjusted R^2
**1 month**			0.30
Anxiety[a]	-0.011**	0.005	
Depression[a]	-0.009**	0.005	
**5 Months**			0.67
HRQoL at month 1	0.789***	0.114	
Musculoskeletal disorder[c]	-0.141***	0.048	
Psychiatric disorder[c]	-0.117**	0.050	
Absence in month 5	-0.008***	0.002	
Absence up to 5 months	0.001***	0.000	
**14 months**			0.42
HRQoL at month 8	0.646**	0.239	

HRQoL: health-related quality of life, *BPI:* Brief Pain Inventory. [a] measured using Hospital Anxiety and Depression Scale. [b] questionnaire measuring average pain in the last week. [c] reference group: general and unspecified disorders. * p < 0.1. ** p < 0.05. *** p < 0.01

Appendix S5: Scenario analyses

We explored two scenario analyses to determine if our 24-month imputation assumption had an effect on the CUA results. In Scenario 1, we assumed that the treatment effect on HRQoL merged at the end of the study (at 24-months rather than 15-months). In other words, we imputed HRQoL at 24 months using the mean of the estimated HRQoL at 14 months of all individuals. Scenario 2 involved imputing HRQoL at 24 months using the same value as at 14 months.

Finally, we explored a third scenario analysis using a time horizon of 14 months instead of 24 months. For our base case, we imputed HRQoL values for 24 months for the CCA to allow comparability with the other methods for a 24-month time horizon. However, in order to conduct a true CCA, the time horizon should have only been 14 months, since there was only HRQoL data up to 14 months.

The cost-effectiveness outcomes remained robust to all scenario analyses exploring different assumptions to impute HRQoL data at 24 months, with the exception of Scenario Analysis 3, where assuming a 14-month time horizon resulted in I-MORE being no longer cost-effective compared to O-ACT for SI, MI, and LMM from the limited societal perspective (Table S10-S13).

Table SX. Cost-effectiveness results of scenario analysis using different methods to handle missing health-related quality of life data at timepoint 24. Estimates are presented as means after 5000 bootstrap simulations. Costs in Norwegian Krone. Missing HRQoL imputed using single imputation.

	Base case	Scenario 1[a]	Scenario 2[b]
QALYs O-ACT	1.300	1.308	1.316
QALYs I-MORE	1.319	1.311	1.303
Inc QALYs	0.019	0.004	-0.013
**Healthcare perspective**			
Costs O-ACT	6,218	6,218	6,218
Costs I-MORE	15,577	15,577	15,577
Incremental costs	9,359	9,359	9,359
INMB[c]	-8,786	-9,240	-9,744
**Limited societal perspective**			
Costs O-ACT	71,785	71,785	71,785
Costs I-MORE	70,483	70,483	70,483
Incremental costs	-1,302	-1,302	-1,302
INMB[c]	1,875	1,421	917

HRQoL: health-related quality of life, *O-ACT:* outpatient acceptance and commitment therapy, *I-MORE:* inpatient multimodal occupational rehabilitation, *QALY:* quality-adjusted[1] life year, *INMB*: incremental net monetary benefit. [a]HRQoL at 24 months imputed with the estimated mean at 14 months. [b]HRQoL at 24 months imputed with the same value at 14 months. [c]based on a cost-effectiveness threshold of €30,000 per QALY gained.

Table SXI. Cost-effectiveness results of scenario analysis using different methods to handle missing health-related quality of life data at timepoint 24. Estimates are presented as means after 5000 bootstrap simulations. Costs in Norwegian Krone. Missing HRQoL imputed using multiple imputation.

	Base case	Scenario 1[a]	Scenario 2[b]
QALYs O-ACT	1.278	1.278	1.277
QALYs I-MORE	1.310	1.311	1.312
Inc QALYs	0.032	0.033	0.034
**Healthcare perspective**			
Costs O-ACT	6,240	6,240	6,240
Costs I-MORE	15,571	15,571	15,571
Incremental costs	9,331	9,331	9,331
INMB[c]	-8,380	-8,341	-8,298
**Limited societal perspective**			
Costs O-ACT	71,929	71,929	71,929
Costs I-MORE	70,565	70,565	70,565
Incremental costs	-1,365	-1,365	-1,365
INMB[c]	2,315	2,354	2,398

HRQoL: health-related quality of life, *O-ACT:* outpatient acceptance and commitment therapy, *I-MORE:* inpatient multimodal occupational rehabilitation, *QALY:* quality-adjusted life-year, *INMB*: incremental net monetary benefit. [a]HRQoL at 24 months imputed with the estimated mean at 14 months. [b]HRQoL at 24 months imputed with the same value at 14 months. [c]based on a cost-effectiveness threshold of €30,000 per QALY gained.

Table SXII. Cost-effectiveness results of scenario analysis using different methods to handle missing health-related quality of life data at timepoint 24. Estimates are presented as means after 5000 bootstrap simulations. Costs in Norwegian Krone. Missing HRQoL predicted using linear mixed models.

	Base case	Scenario 1[a]	Scenario 2[b]
QALYs O-ACT	1.269	1.275	1.281
QALYs I-MORE	1.268	1.263	1.257
Inc QALYs	-0.001	-0.012	-0.024
**Healthcare perspective**			
Costs O-ACT	6,232	6,232	6,232
Costs I-MORE	15,576	15,576	15,576
Incremental costs	9,344	9,344	9,344
INMB[c]	-9,363	-9,692	-10,058
**Limited societal perspective**			
Costs O-ACT	71,869	71,869	71,869
Costs I-MORE	70,558	70,558	70,558
Incremental costs	-1,311	-1,311	-1,311
INMB[c]	1,293	963	597

HRQoL: health-related quality of life, *O-ACT:* outpatient acceptance and commitment therapy, *I-MORE:* inpatient multimodal occupational rehabilitation, *QALY:* quality-adjusted life year, *INMB*: incremental net monetary benefit. [a]HRQoL at 24 months imputed with the estimated mean at 14 months. [b]HRQoL at 24 months imputed with the same value at 14 months. [c]based on a cost-effectiveness threshold of €30,000 per QALY gained.

Table SXIII. Scenario analysis results of using different methods to impute missing health-related quality of life data in a cost-utility analysis for I-MORE vs. O-ACT using a 14-month time horizon. Estimates are presented as means after 5000 bootstrap simulations. Costs in Norwegian Krone.

	CCA[a]	SI	MI	LMM
QALYs O-ACT	0.689	0.744	0.732	0.739
QALYs I-MORE	0.719	0.765	0.763	0.733
Inc QALYs	0.031	0.021	0.032	-0.006
**Healthcare perspective**				
Costs O-ACT	4,388	4,246	4,270	4,267
Costs I-MORE	13,452	14,229	14,225	14,227
Incremental costs	9,064	9,982	9,955	9,961
INMB[b]	-8,139	-9,359	-9,008	-10,135
**Limited societal perspective**				
Costs O-ACT	42,666	39,360	39,471	39,417
Costs I-MORE	39,789	43,120	43,182	43,183
Incremental costs	-2,878	3,761	3,711	3,766
INMB[b]	3,802	-3,137	-2,764	-3,940

I-MORE: inpatient multimodal occupational rehabilitation, *O-ACT:* outpatient acceptance and commitment therapy, *CCA:* complete case analysis, SI: single imputation, *MI:* multiple imputation, *LMM:* linear mixed models, *QALY:* quality-adjusted life year, *INMB*: incremental net monetary benefit. [b]CCA sample size is 42 instead of 159. [b]based on a cost-effectiveness threshold of €30,000 per QALY gained.
